# Revisiting the interpretation of axon diameter mapping using higher-order signal representations

**DOI:** 10.1162/IMAG.a.1080

**Published:** 2026-01-09

**Authors:** Bradley G. Karat, Jamie Wren-Jarvis, Erika P. Raven, Ali R. Khan, Derek K. Jones, Marco Palombo, Jelle Veraart

**Affiliations:** Robarts Research Institute, Western University, London, ON, Canada; Centre for Functional and Metabolic Mapping, Western University, London, ON, Canada; Center for Biomedical Imaging, Department of Radiology, NYU Grossman School of Medicine, New York, NY, United States; Institute for Translational Neuroscience, NYU Grossman School of Medicine, New York, NY, United States; Cardiff University Brain Research Imaging Centre (CUBRIC), School of Psychology, Cardiff University, Cardiff, United Kingdom; School of Computer Science and Informatics, Cardiff University, Cardiff, United Kingdom

**Keywords:** axon diameter, diffusion MRI, high b-value, glia, spherical mean, spherical variance

## Abstract

Diffusion-weighted Magnetic Resonance Imaging (dMRI) has emerged as an imaging modality of interest to measure axon diameters noninvasively. The previously observed *b*^-½^ power law scaling suggests that high *b*-value dMRI signals originate from water confined within “stick” geometries, representing impermeable cellular processes. A key assumption is that any deviation from this power law at high *b-*values—modeled as a non-zero perpendicular intracellular diffusivity—must be specifically axonal in origin. Recent developments in axon diameter mapping build upon such assumptions, thereby neglecting the possibility that other cellular structures, such as glial processes, may also exhibit similar “stick”-like characteristics. This explorative study investigates the validity of axon diameter mapping by evaluating its robustness to experimental variation. In particular, it compares the mapping of the axon diameter using the zeroth- (spherical mean) and second-order (spherical variance) rotationally invariant spherical harmonic (RISH) features. As a condition for validity, axon diameter should be robust to such variations in RISH order. A novel log-linear estimator with a closed-form solution for computationally efficient axon diameter mapping is introduced, which can be applied with a minimum of two high *b-*value measurements. Using this estimator, it was observed that axon diameter measurements vary with RISH order, suggesting that high *b*-value signals from non-axonal cellular sources may confound axon diameter mapping. Monte Carlo simulations show that such dependence on RISH order could be explained by the presence of glial processes. Overall, these results highlight the need for caution in the interpretation of dMRI-derived “*axon*” diameter.

## 1. Introduction

Neural conduction velocity is determined by axon diameter, making it a key factor in brain function (Boyd & Kalu, 1979; Drakesmith et al., 2019; Schmidt & Knösche, 2019; Waxman, 1980). The ability to map axon diameters non-invasively and robustly would greatly advance the study of structure-function relationships across the lifespan and in health and disease. Indeed, axon diameter has been shown to be altered in a variety of diseased states. For example, regional enlargement of axons in the anterior horn of the spinal cord and in the somatic motor nuclei in the brainstem have been observed in motor neuron disease (Carpenter, 1968). In Amyotrophic Lateral Sclerosis (ALS), it is believed that the smaller diameter axons are relatively spared, while the larger diameter axons (i.e., type alpha) are damaged, and that the diameter of the initial axonal segments is increased (Heads et al., 1991; Kiernan & Hudson, 1991; Sasaki & Maruyama, 1992). Axon diameter mapping, by non-MR means, has also been of great interest in a variety of neurodevelopmental disorders. Electron microscopy in the corpus callosum of individuals with autism spectrum disorder revealed a reduction in axon diameter, with an increase in the percent of smaller diameter axons and an attenuation of axons with larger diameters (Wegiel et al., 2018). In a mouse model of Angelman Syndrome which captures the phenotype of microcephaly, Judson et al. (2017) observed largely intact myelination of corpus callosum and sciatic nerve axons with marked reductions in axon caliber and subsequent deficits in nerve conduction. An MRI-based study on 22q11.2 deletion syndrome, which is associated with high rates of schizophrenia and autism, hypothesized that abnormal structural connectivity may be explained by axons which are densely packed yet have disproportionately small diameters (Raven et al., 2023). Finally, differences in axon diameter have been noted between sexes and across healthy aging in rodents. Males have generally been found to have larger axon diameters than females (Pesaresi et al., 2015; Zhou et al., 2018), and older mice were found to have larger axon diameters and a smaller number of axons per unit area then their younger counterparts (Stahon et al., 2016). Thus, reliably mapping axon diameter *in vivo* would be indispensable as a biomarker and as a tool to understand the organization principles of the brain in health and disease.

Diffusion MRI (dMRI) is sensitive to the diffusion of water at a micron-scale, which is commensurate with tissue microstructure (Beaulieu, 2002; Jones, 2011; Le Bihan & Breton, 1985; Stejskal & Tanner, 1965). This provides a remarkable opportunity to characterize structures which exist well below the spatial resolution of the imaging modality (Novikov, 2021; Stanisz et al., 1997). Axon diameter mapping using dMRI has been pursued by various researchers during the past 15 years (Alexander et al., 2010; Assaf et al., 2008, 2013; Barazany et al., 2009; Lee et al., 2020; Ong & Wehrli, 2010; Veraart et al., 2020; Xu et al., 2014). The significant overestimation of axon calibers in the earliest results were subject to controversy (Innocenti et al., 2015). However, driven by advances in gradient designs and novel insights in biophysical modeling, we are now witnessing a promising revival in the development, validation, and ultimately clinical use of axon diameter mapping (Huang et al., 2015; Veraart et al., 2020). Two key features are (a) the use of high *b*-values to suppress extra-cellular signals (McKinnon et al., 2017; Veraart et al., 2019) and (b) spherical mean techniques to mitigate the impact of axonal dispersion (Anderson, 2005; Kaden et al., 2016; Novikov, Veraart, et al., 2018; Reisert et al., 2017).

Axon diameter mapping, similar to any other biophysical modelling approach, is built upon a series of assumptions regarding the microstructure or its impact on the dMRI signal (Jelescu et al., 2020; Novikov, Kiselev, et al., 2018; Panagiotaki et al., 2012). For example, in a strategy proposed by Veraart et al. (2020) it is assumed that the diffusion in the extra-axonal space is hindered, not restricted, and sufficiently fast to the extent that its resulting signal contributions are negligible at *b*-values of 6 ms/µm^2^ and higher, in any direction. Dedicated studies have provided corroborating data, showing that the dMRI signal at such high *b*-values can be attributed to water confined in micrometer-thin and impermeable cellular structures (McKinnon et al., 2017; Veraart et al., 2019). Often, it is further assumed that dMRI signal at high *b*-values is specific to axons. It is critical to validate such assumptions to avoid mis- or overinterpretation of findings. In this explorative study, we aim to challenge the assumption of axonal-specificity of strongly diffusion-weighted signals by putting it to a tailored test.

The validation of biophysical modeling is inherently challenging, typically because of the lack of a ground truth (Jelescu et al., 2020). Microstructural properties can be extracted through histology, but such properties might be impacted by fixation effects (Aboitiz et al., 1992), have limited field-of-view relative to the voxel sizes of dMRI (Veraart et al., 2020), or lack a one-to-one correspondence to the MR features that are validated. Notorious examples include the difference between the MR-derived “signal fraction” and the nominal volume fractions of a microstructural compartment (Jespersen et al., 2007), or the discrepancy between an average radius of a distribution of axons and the corresponding effective MR radius (Alexander et al., 2010; Burcaw et al., 2015; Jelescu & Budde, 2017; Jelescu et al., 2016; Veraart et al., 2020). Monte Carlo simulations can be used to model the dMRI signal from realistic tissue geometries (Callaghan et al., 2020; Ianuş et al., 2021; Palombo et al., 2016, 2019). Such approaches provide, for example, an avenue to study the impact of axonal undulations or curvature on the axon diameter mapping (Lee et al., 2020, 2024), or the impact of intra-/extra-axonal exchange (Hill et al., 2021). However, simulations often have limited generalizability as they tend to be constrained to single cell types, thereby ignoring the cellular complexity and diversity of biological tissue. In this study, we aim to supplement any of these validation approaches and to gain more confidence in the validity of MR-based axon diameter mapping by evaluating its robustness to experimental variation (Kiar et al., 2024). Indeed, if valid, estimates of axon diameters must be robust to variations in experimental conditions (e.g., SNR) and design choices (e.g., *b*-values).

In recent years, axon diameter mapping has been advanced by fitting a biophysical model to the *spherical mean* of, minimally, data collected at two ultra-high *b*-value shells (McKinnon et al., 2017; Veraart et al., 2019, 2020). The spherical mean is *de facto* the zeroth order rotationally invariant spherical harmonic (RISH) feature (Mirzaalian et al., 2016). The spherical mean is uniquely invariant to the orientation distribution function (ODF) of the underlying axons, thereby enabling axon diameter mapping in regions with complex fiber configurations, while mitigating the effect of axonal dispersion (Andersson et al., 2022; Callaghan et al., 1979; Jespersen et al., 2013; Kaden et al., 2016). However, following the same theory, higher-order RISH features can be used to derive the axon diameter, albeit limited to voxels with minimal axonal dispersion (Pizzolato et al., 2022). Indeed, the signal-to-noise ratio (SNR) of such higher-order RISH features is inversely proportional to the degree of dispersion, with no measurable signal when the diffusion is fully isotropic (Novikov, Veraart, et al., 2018). While at first glance, this dependency disfavors its further use, we aim to use the higher-order RISH features to test the validity of axon diameter mapping and its underlying assumptions (Callaghan et al., 1979; Kroenke et al., 2004; McKinnon et al., 2017; Veraart et al., 2019). As a minimal condition for validity, axon diameter mapping must be robust to variations in the RISH order. Indeed, any inconsistencies would suggest the presence of an additional restricted signal compartment of spheres and/or a completely orientationally dispersed set of cylindrical geometries (Pizzolato et al., 2023).

The microstructure of the white matter is complex and diverse in its cellular composition. The potential presence of spherical compartments of restricted diffusion, for example, the so-called *dot compartment*, has been previously conjectured, but without conclusive results in the living human brain (Dhital et al., 2018; Panagiotaki et al., 2012; Tax et al., 2020; Veraart et al., 2019). Alongside axons, one can identify various cell types including oligodendrocytes, astrocytes, and microglia, collectively referred to as *glial cells* in the white matter. Glial cells are a dominant cell type in the white matter and are critical for the modeling and maintenance of myelin, energy supply, and waste clearance. The morphological features of glial cells are known to be dynamic, but overall, they are characterized by a collection of *dispersed* processes. These processes are micrometer-thin, but their diameters exceed, on average, the diameter of axons (Liewald et al., 2014; Savtchenko et al., 2018). Critically, glial processes are known to be more isotropically distributed, even in regions with well-aligned axons (Jessen, 2004; Savtchenko et al., 2018).

In this study, we will (a) evaluate the internal consistency of axon diameter mapping from in vivo human data by varying the order of the representation of the diffusion-weighted signal at high *b*-value, and (b) evaluate using Monte Carlo simulations whether the potential lack of consistency can be explained by the presence of glial processes. In addition, this study introduces a novel log-linear estimator for computationally efficient axon diameter mapping with a closed-form solution.

## 2 Methods

### 2.1 MRI data

This study presents a secondary analysis of previously published test/retest diffusion MRI data of 5 healthy adult volunteers that were collected under the approval of the Cardiff University School of Psychology Ethics Committee at the Cardiff University Brain Research Imaging Centre (CUBRIC) (Veraart et al., 2021). All data were acquired on a Siemens Connectom 3T MRI scanner using a 300 mT/m gradient coil and 32-channel receiver coil. Test and retest data were collected on the same day, only separated by a short break and re-positioning of the subjects. All diffusion-weighted images were acquired with a multi‐band blipped‐CAIPI accelerated (SMS = 2) EPI sequence, without Partial Fourier encoding, but with GRAPPA acceleration (R = 2); TR/TE: 3500/66 ms and an in-plane spatial resolution of 2.5 × 2.5 mm^2^ and slice thickness of 2.5 mm for 54 slices. In addition to 23 non-diffusion-weighted images, diffusion-weighted images were obtained by applying diffusion gradients with Δ/δ = 30/15 ms and varying gradient amplitude with a maximum of 273 mT/m. In total, we acquired 450 diffusion-weighted images distributed across the following *b*-shells: 0.5, 1, 2.5, 6, and 30 ms/µm^2^. The corresponding number of gradient directions, uniformly distributed on a sphere, were 30, 30, 30, 120, and 240, respectively. Ten non-diffusion-weighted images with reversed phase encoding were acquired to perform susceptibility‐induced geometrical distortion correction.

### 2.2 Image preprocessing

Diffusion MRI data were preprocessed using an *in-house* workflow prior to further analysis to minimize the impact of imaging artifacts. The workflow included detection and removal of signal outliers and corrections for Gibbs ringing (Kellner et al., 2015; Sairanen et al., 2018), subject motion, and susceptibility‐ and eddy current distortions (Andersson & Sotiropoulos, 2015). Gradient amplitude non-uniformities were corrected by scaling the *b*-values voxel-by-voxel using an *in-house* pipeline that leverages a scanner-specific proprietary gradient amplitude non-uniformity map (Rudrapatna et al., 2021). The noise level was estimated for Rician bias correction from the DW images with *b* ⩽ 1 ms/µm^2^ using MPPCA (Veraart et al., 2016).

### 2.3 Extracting RISH features

First, per *b*-value, we estimated the spherical harmonic coefficients (Lmax = 6) of shelled dMRI data with a Maximum Likelihood estimator using a Rician likelihood function and an *a priori* estimated noise level (Sijbers et al., 1998). We then computed the zeroth and second-order Rotationally-Invariant Spherical Harmonic (RISH) features as the norm of the respective SH coefficients for all *b*-values (cf. *S*_0_ and *S*_2_ from Novikov, Veraart, et al., 2018) and normalized them by the non-diffusion weighted signal. In line with the works of Pizzolato et al. (2022) and Raven et al. (2023), we will refer to the zeroth and second-order RISH features as the “*spherical mean*” (SM) and “*spherical variance*” (SV), respectively.

### 2.4 Standard model imaging

We use Standard Model Imaging (SMI) to estimate the following biophysical model parameters from the RISH features for all *b*-values up to *b* = 6 ms/µm^2^: intra-cellular signal fraction *f*, intra-cellular parallel diffusivity *$D_{C}^{\left|\right|}$ *, intra-cellular coherence index *p*_2_, and extra-cellular diffusivities using a nonlinear least-squares estimator (Novikov, Kiselev, et al., 2018). Note that the *b* = 30 ms/µm^2^ was omitted due to its sensitivity to perpendicular intracellular diffusivity, thereby violating the SMI assumptions.

### 2.5 Fiber tractography

The MRtrix3.0 package was used for estimating the fiber Orientation Distribution Function (fODF; *L*_max_ = 8) from the dMRI data for *b* = 30 ms/µm^2^ using constrained spherical deconvolution in a spherical harmonics basis (Tournier et al., 2010, 2019). The signal kernel was estimated using the algorithm of Tournier et al. (2013). The highest b-shell was used for improved tractography (Yu et al., 2025).

### 2.6 Study-specific template

We generated a study-specific template space using an iterative image registration of the zeroth-order RISH maps of all subjects. The template was created using MRtrix’s *population_template* command (Tournier et al., 2019) including *b* = 6 ms/µm^2^ diffusion data from each subject and session. As part of this template generation, the transformations, that is, affine transformation and warp field, that map individual subject data with the template are stored. We applied these transformations to the subject-specific RISH and fODF maps to align all data in a common unbiased template.

### 2.7 Tract segmentation

We first derived the main fiber orientations, or *peaks*, of the subject-averaged fODFs in template space. These peaks then serve as the input for TractSeg, an open-source pre-trained Convolutional Neural Network for fast and accurate white matter bundle segmentation from dMRI data (Wasserthal et al., 2018; https://github.com/MIC-DKFZ/TractSeg).

### 2.8 Tractometry

We performed along-tract profiling of the subject-specific RISH features in the population space (Yeatman et al., 2018). We initially limited our study to the corticospinal tract (CST). The CST was divided into 98 segments, and the RISH features were averaged within individual segments. The TractSeg toolbox was used for this analysis (Wasserthal et al., 2018).

### 2.9 Axon diameter mapping

Under the approximation of $erf\left(\sqrt{bD_{c}^{\left|\right|}}\right)$ = 1 for *b*-values of minimally *b* = 6 ms/µm^2^ (Veraart et al., 2019), we here adopt a single-compartment model of restricted diffusion in a cylinder with radius *r*, previously introduced in (Veraart et al., 2020):

$$\bar{S}\left(b\right)\;=\;\beta \frac{S_{c}^{⊥}(r\;|\;g,\delta )}{\sqrt{b}}.$$ (1)

$\bar{S}\left(b\right)$ represents the normalized spherical mean signal, $\beta =\sqrt{\frac{f}{D_{C}^{\left|\right|}}}$ is a prefactor that depends on the intra-cellular signal fraction *f*, and parallel apparent diffusivity $D_{c}^{\left|\right|}$ , $S_{c}^{⊥}(r\;|\;g,\delta )$ is the restricted signal decay in a cylinder perpendicular to its main axis for a given gradient magnitude g and duration  δ . We further adopt the Neumann model (Neuman, 1974):

$$S_{c}^{⊥}(r|g,\delta )=\exp(-\frac{7}{48}\frac{g^{2}\delta r^{4}}{D_{0}}),$$ (2)

with $D_{0}$ the intrinsic diffusivity of the axoplasm, set fixed to 2.5 µm^2^/ms. This model is less general than the model of Van Gelderen et al. (1994) because its use is limited to scan regimes with $\delta \gg \frac{r^{2}}{D_{0}}$. However, when using the Neumann model, we can linearize the model using a log transformation, thereby enabling an efficient computation of the effective MR radius r:

$$log\left(\sqrt{b}\;\bar{S}\left(b\right)\right)\;=\;log\left(\beta \right)\;-\;\kappa \left(g,\delta \right)r^{4},\;$$ (3)

with $\kappa \left(q,\delta \right)=\frac{7}{48}\frac{g^{2}\delta }{D_{0}}$. The application of the single-compartment model is limited to the “*high b*” regime to minimize signal contributions of the extra-axonal water (Veraart et al., 2019; McKinnon et al., 2017). Therefore, axon diameter mapping is here limited to dMRI data acquired with *b* = 6 and 30 ms/µm^2^. In case of two such *b*-values, we can estimate the effective MR radius as follows:

$$r^{4}=\;\frac{log\left(\frac{\sqrt{b_{1}}\;\bar{S}\left(b_{1}\right)}{\sqrt{b_{2}}\bar{S}\left(b_{2}\right)}\right)}{\kappa \left(g_{2},\;\delta_{2}\right)-\kappa \left(g_{1},\;\delta_{1}\right)}.\;$$ (4)

Note that we further assumed that $bD_{C}^{\left|\right|}\gg \frac{7}{48}\frac{g^{2}\delta r^{4}}{D_{0}}$ (see also Pizzolato et al. (2023) and Veraart et al. (2020)). Although the model was initially introduced for the spherically-averaged signal, it can be extended to higher order rotational invariants. Indeed, Anderson (2005) derived higher-order RISH models for arbitrary intra-cellular radial diffusivity $D_{C}^{⊥}\;$ and Jensen et al. (2016) developed Fiber Ball Imaging as a special case with $D_{C}^{⊥}=0$ . Here, we adopt the work of Anderson (2005) to develop a mode for axon diameter mapping from the second order RISH ${\bar{S}}_{\sigma }\left(b\right)$:

$${\bar{S}}_{\sigma }\left(b\right)\;=\;p_{2}\gamma \left(3-2bD_{C}^{\left|\right|}\right)\frac{S_{c}^{⊥}(\rho \;|\;g,\delta )}{\sqrt{b^{3}}},$$ (5)

with $\gamma =\frac{5}{8}\sqrt{\frac{f}{{\left(D_{C}^{\left|\right|}\right)}^{3}}}$ and $p_{2}$ the intra-cellular coherence index. Note that compared to Anderson (2005), we substituted $exp\left(-bD_{C}^{⊥}\right)$ by $S_{c}^{⊥}(r\;|\;g,\delta )$ . This generalization does not introduce new model assumptions, but $D_{C}^{\left|\right|}$ becomes an additional model parameter to be estimated. Note that we estimated $D_{C}^{\left|\right|}$ a priori using Standard Model Imaging (Novikov, Veraart, et al., 2018). Similar to Eq [3], one can estimate $r^{4}$ as follows if exactly two *b*-values are available:

$$r^{4}=\frac{log\left(\sqrt{b_{1}^{3}}\left(3-2b_{2}D_{C}^{\left|\right|}\right)\;\;{\bar{S}}_{\sigma }\left(b_{1}\right)\right)-log\left(\sqrt{b_{2}^{3}}\;\left(3-2b_{1}D_{C}^{\left|\right|}\right){\bar{S}}_{\sigma }\left(b_{2}\right)\right)}{\kappa \left(g_{2},\;\delta_{2}\right)-\kappa \left(g_{1},\;\delta_{1}\right)}.$$ (6)

While the effective MR radius can be derived voxel-wise, we here limit further analysis to segments as it has been shown to be a more precise and reproducible approach (Veraart et al., 2021).

### 2.10 Statistical analysis

We first tested the hypothesis that the effective MR radii, as derived from SM and SV, are not significantly different within our subject cohort. We used a paired two-sided t-test with a significance level of 0.05. To improve sensitivity, we averaged the effective MR radii across their repeated measurements. The analysis was performed per tract segment (cf. Veraart et al., 2021).

We quantified the test-retest variability (TRV) as a metric of repeatability of effective MR radius measurements. The TRV was evaluated for each tract segment as follows:

$$TRV=\frac{1}{N}\sum_{i=1}^{N}\frac{\sqrt{\pi }}{2}(2|\frac{a-b}{a+b}|)\times 100,$$

with *a* and *b* representing repeated measurements and *N* the number of subjects. We also quantified the inter-subject variability using the coefficient of variation (CoV) per tract segment. More specifically, we compute the ratio between the standard deviation of metrics of interest across subjects and their subject-averaged value. This analysis was performed on the estimates of MR effective radii and on the RISH features themselves.

Finally, we compute Lin’s Concordance Correlation Coefficient $\rho_{c}$ and the coefficient of accuracy (Lin & Torbeck, 1998) to measure *agreement* between the along-tract profiles of the effective MR radii, as derived from SM and SV.

### 2.11 Monte Carlo simulations

The impact of an isotropic cellular compartment in addition to the axonal compartment on the estimation of the effective axon radius was investigated using Monte Carlo simulations of spin diffusion within synthetic cellular substrates from real microscopy reconstructions. We reconstructed the three-dimensional surfaces of 10 microglia (Diniz et al., 2016), 10 astrocytes (Canchi et al., 2017; Kovács & Pál, 2017; Papageorgiou et al., 2011; Savtchenko et al., 2018), and 10 oligodendrocytes (Chan et al., 2013; Fannon et al., 2015) from the neuromorpho.org database. We generated synthetic diffusion-weighted MRI signals using the same gradient directions, b-values, and Δ/δ as used in the MRI protocol, using 10^4^ walkers uniformly distributed within each glial cell/axon, diffusivity fixed at 2.5 µm^2^/ms and fixed-length off-lattice step of 0.40 μm (Hall & Alexander, 2009). We additionally used the matrix formalism for diffusion signal attenuation within a dispersed set ($p_{2}$ =0.7) of fully restricted cylinders to model axonal signal (Ianuş et al., 2016). This approach has been shown to be more accurate to test axon diameter mapping than using axonal morphology derived from microscopy as beading and undulations impact the measurements (Lee et al., 2024). We did not stimulate the contribution of the extracellular compartment as it fully decayed at *b* = 6 ms/µm^2^ (Veraart et al., 2019) and further assumed exchange to be negligible at diffusion times ≤45 ms (Pfeuffer et al., 1998; Yang et al., 2017).

## 3 Results

### 3.1 Qualitative assessment of RISH maps

**Fig. 1.**
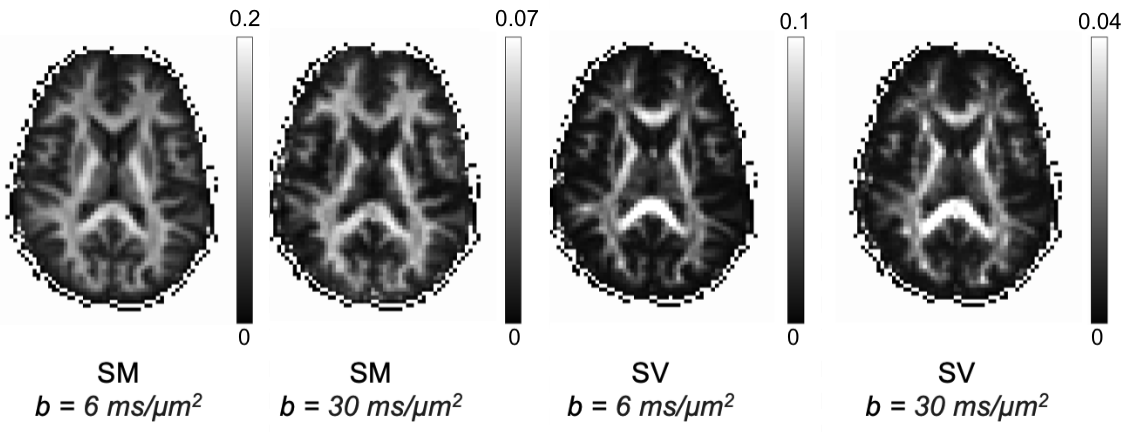
Example normalized Spherical Mean (SM) and Spherical Variance (SV) maps from *b*-value shells of 6 and 30 ms/μm^2^.

Figure 1 depicts the maps of the zeroth and second order RISH features (SM and SV, respectively) at high *b*-values. Both SM and SV maps show strong contrast between white matter and surrounding tissue. While there is some heterogeneity within the white matter in the SM maps, we do not observe a loss of signal in areas that are notorious for their crossing fibers. In contrast, but much like fractional anisotropy maps, the SV signal is more sensitive to the underlying fiber architecture with values dropping to zero in regions with crossing fibers or axonal dispersion.

### 3.2 Along-tract profile of the effective MR radius

**Fig. 2.**
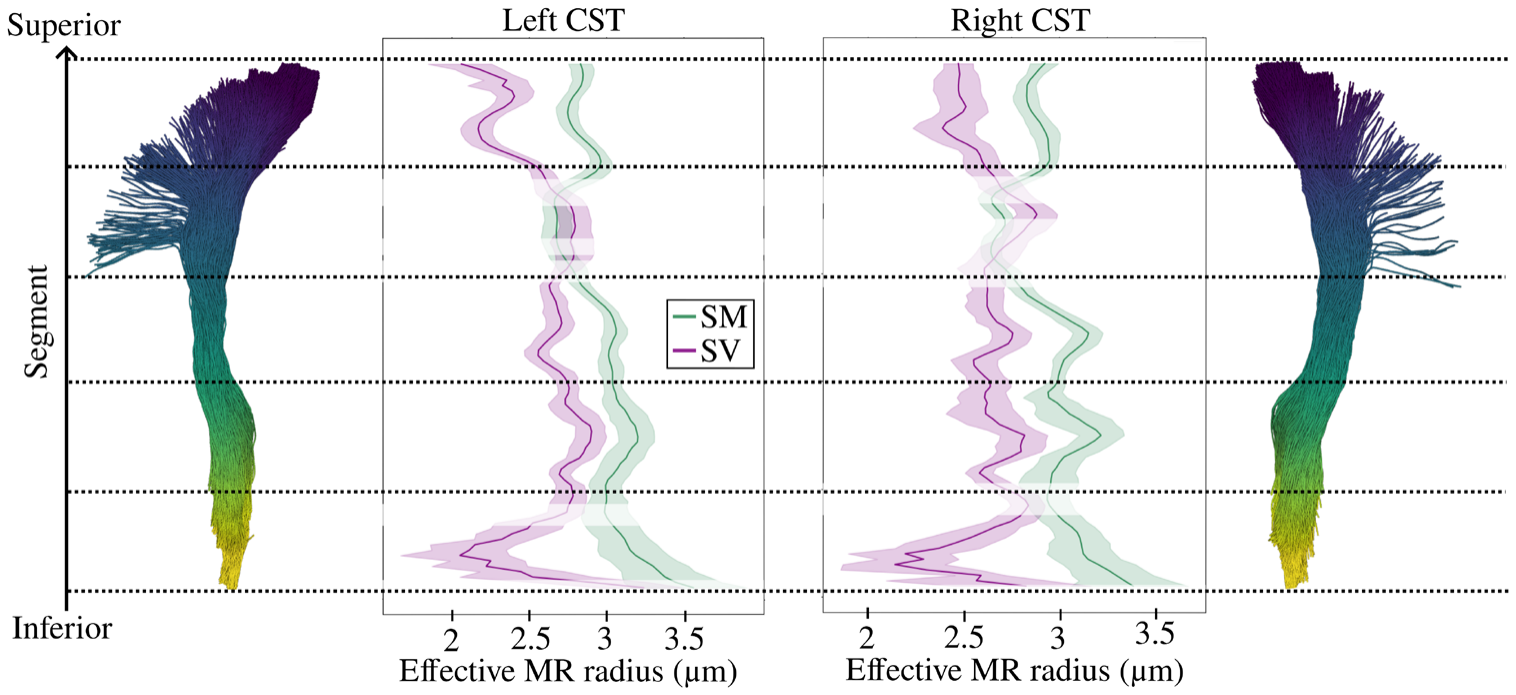
Characterization of the effective MR radius across subjects per segment in the left and right cortico-spinal tract (CST). The tract is split into 98 segments, where 1 is the most anatomically superior segment and 98 the most inferior, as shown in the tractography reconstruction of the CST in the middle. The mean of the effective MR radius computed from the spherical Mean (SM) is marked with the solid green line and the spherical variance (SV) is marked with the solid purple line. The 95% confidence interval (CI) is included for both the SM and SV plots. Results of the paired t-test comparing the effective MR radius values from SM and SV measurements within each segment are indicated. Significant differences (*p* < 0.05) from a paired t-test comparing the effective MR radius values from SM and SV measurements are demarcated by the regions with no translucent overlay, while the regions with a white translucent overlay indicate no significant difference between the SM and SV for that segment. In Supplementary Figure S1, we test the generalizability of these results to other WM fiber tracts.

In Figure 2 we show the subject-averaged effective MR radii along the cortico-spinal tract (CST) for both hemispheres, as derived from the zeroth and second order RISH features (referred to as $r_{SM}$ and $r_{SV}$ , respectively). Values for $r_{SV}$ tend to be lower than $r_{SM}$ . The trends of the average $r_{SM}$ and $r_{SV}$ per segment seen in Figure 2 are consistent across hemispheres. The average across segments for $r_{SM}$ is 2.943 ± 0.241 μm in the right CST and 2.959 ± 0.271 μm in the left CST. As for $r_{SV}$ , the average value is 2.630 ± 0.254 μm in the right CST and 2.602 ± 0.322 μm in the left CST. The percentage difference between SM/SV across segments varies between 0.36% and 35.67%, with a median value of 11.04% in the left CST and varies between 0.49% and 33.61% with a median value of 12.12% in the right CST. We further observe a weak correlation between $p_{2}$ and the relative difference between $r_{SM}$ and $r_{SV}$ , with a Spearman’s Correlation Coefficient of 0.22 (Supplementary Fig. S2). All radii were estimated using the log-linear estimator. However, the percentage difference in the estimation of effective MR radii across estimators is relatively small. The median percentage difference in the estimation of $r_{SM}$ and $r_{SV}$ using log-linear vs. nonlinear estimators is 0 and 1.47%, respectively, if Neumann’s model is used, or 1.81% and 0.69%, respectively, if the nonlinear estimator uses Van Gelderen’s model. The comparison includes all subjects, sessions, and segments of the CST.

### 3.3 Within- and between-subject variability

The TRV was used to evaluate the repeatability of effective MR radius measurements and the RISH features. For $r_{SM}$ , the TRV is 2.96% in the left CST and 2.95% in the right CST. For $r_{SV}$ , the TRV is slightly higher with 4.66% in the left CST and 5.87% in the right CST. The test-retest reproducibility of the radii is, thus, consistent across both hemispheres. We further quantify the TRV, averaged across hemispheres, for the SM and SV signals at *b* = 6 and 30 ms/µm^2^. The average TRV of SM is 2.57% and 2.63% for *b* = 6 and 30 ms/µm^2^, respectively. For SV, the average TRV is 4.17% and 5.97% for the respective *b*-values. The inverse of the TRV is a proxy of the SNR of this measurement.

The CoV was used to quantify the inter-subject variability of the effective MR radius within segments of the CST. For $r_{SM}$ , the values have a CoV percentage of 6.06% in the left CST and 4.96% in the right CST. For $r_{SV}$ , the CoV percentage is 6.89% in the left CST and 7.27% in the right CST.

### 3.4 Concordance analysis

Per subject, we measure agreement of the estimated effective MR radius between the test-retest data (separately for SM and SV) and between SM and SV for test and retest separately using Lin’s Concordance Correlation Coefficient, $\rho_{c}$ and the coefficient of accuracy (Table 1). The average $\rho_{c}$ between the test-retest for $r_{SM}$ is 0.76 with a mean coefficient of accuracy of 0.91. The average $\rho_{c}$ between the test-retest for $r_{SV}$ is 0.58 with a mean coefficient of accuracy of 0.91. The high coefficient of accuracy highlights that any differences in $\rho_{c}$ for $r_{SM}$ and $r_{SV}$ are driven by the precision of the measurements. As well, the higher $\rho_{c}$ for the SM suggests greater reliability in measuring the effective MR radius as compared to the SV. For both the test and retest data, $\rho_{c}$ is much lower when measuring the agreement between $r_{SM}$ and $r_{SV}$ with values of 0.04 and -0.02, respectively. In this comparison, the coefficient of accuracy is significantly lower, highlighting the presence of systematic differences between the effective MR radius estimated with the SM and SV.

**Table 1.** The mean and standard deviation (SD) of Lin’s concordance correlation coefficient ($\rho_{c}$) and the coefficient of accuracy for the estimated effective MR radius between the test-retest data (separately for SM and SV) and between SM and SV (separately for test and retest data).

Comparison	Mean (SD) of $\rho_{c}$	Mean (SD) of the coefficient of accuracy
SM (test-retest)	0.76 (0.17)	0.91 (0.14)
SV (test-retest)	0.58 (0.14)	0.91 (0.07)
SM-SV (test)	0.04 (0.18)	0.49 (0.13)
SM-SV (retest)	-0.02 (0.08)	0.44 (0.07)

### 3.5 Robustness to set diffusivities

**Fig. 3.**
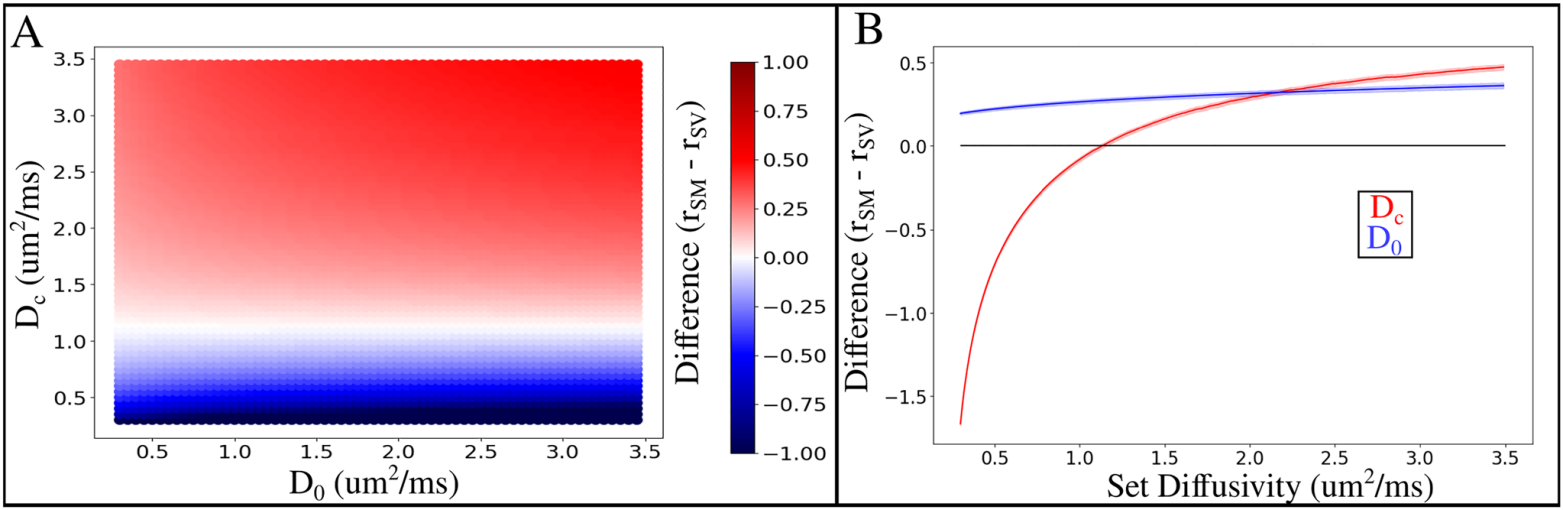
Dependency of the difference of effective MR radius between SM and SV on varying fixed diffusivities ($D_{0}$ and $D_{c}$). (A) Difference between SM and SV for varying $\;D_{0}$, $D_{c}$ pairs. The average difference was taken across sampling points, subjects, and sessions for each $D_{0}$, $D_{c}$ pair. (B) Using the diffusivity values of the current study. For each chosen $D_{c}$ (red), $D_{0}$ was fixed at 2.5 um^2^/ms. For each chosen $D_{0}$ (blue), $D_{c}$ was chosen to be the value obtained from the SMI. Black horizontal line depicts where the difference in radius between SM and SV equals 0.

Figure 3 depicts the difference in the effective MR radius between the SM and SV as a function of fixed $D_{0}$ and $D_{C}^{\left|\right|}$ (referred to here as $D_{c}$). The difference in the estimates can be seen to be largely dependent on $D_{c}$, with minimal change across $D_{0}$ (Fig. 3A). The difference between SM and SV is minimized at a $D_{c}$ of 1.15 µm^2^/ms across all $D_{0}$. With a $D_{c}$ greater than 1.15 µm^2^/ms, the effective radius estimated from SM becomes increasingly greater than the SV estimated effective radius. Conversely, at a $D_{c}$ less than 1.15 µm^2^/ms, the SV effective radius becomes greater than the SM effective radius. The range of $D_{c}$ estimated from SMI in the current study was between [1.95, 2.63] µm^2^/ms, existing in the upper quadrants of the difference plot in Figure 3A. Figure 3B depicts the difference between SM and SV using the diffusivity values from SMI. It can be seen that using these $D_{c}$ values, no set $D_{0}$ minimizes the difference between the SM and SV estimated MR radius (blue line; Fig. 3B). For a fixed $D_{0}$ of 2.5 um^2^/ms, the difference between SM and SV is minimized at a $D_{c}$ of 1.15 µm^2^/ms, as noted in Figure 3A.

### 3.6 Monte Carlo simulations

**Fig. 4.**
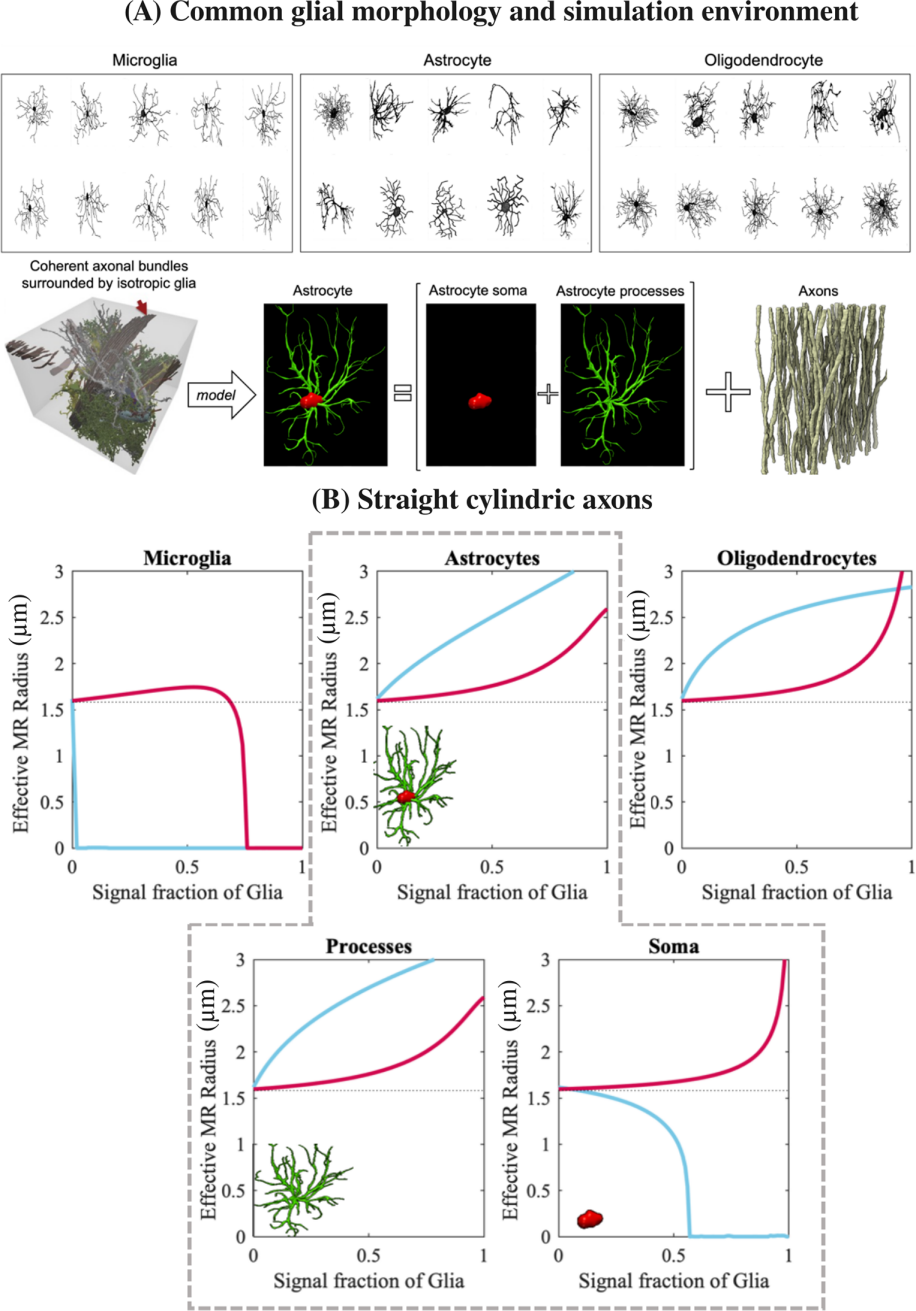
Impact of realistic glia morphology (isotropic cellular compartment) on the effective axon radius derived from the spherical mean (SM) and spherical variance (SV) in the presence of a coherent axon bundle using Monte Carlo simulations of spin diffusion. (A) Variety of microglia, astrocyte, and oligodendrocyte morphology as derived from real microscopy reconstructions. The synthetic substrate includes a coherent axon bundle surrounded by a particular glial cell. (B) SM (blue) and SV (red) derived effective MR radius as a function of glia signal fraction in a substrate of straight cylindrical axons. The bottom row simulates the effect of astrocytic processes vs soma separately. The grey dotted line depicts the ground-truth effective radius.

We show the effect of glia (microglia, astrocyte, and oligodendrocyte) on the effective MR radius derived from the SM and SV in Figure 4. Figure 4A shows some examples of realistic glia morphology that were used in the simulation and an example synthetic substrate including a coherent axon bundle (Andersson et al., 2020). Figure 4B depicts the effective MR radius measured using the SM or SV of the simulated signal in the presence of varying glia signal fractions. In the absence of a glia contribution, both axon diameter estimates are accurate, regardless of the use of SM or SV. However, we show that discrepancies appear, even for small fraction of glial contributions, but the effects vary across the glial cell types. First, axon diameter mapping using SM is not reliable if a large fraction of spherical compartments, relative to processes, contributes to the diffusion-weighted signal. We observe these trends in the presence of microglia or large somas of astrocytes. Axon radii estimated from SV are more robust as such spherical contributions are cancelled out. Second, the trends for the astrocytes and oligodendrocytes are similar to each other, with the effective MR radius measured with the SM and SV increasing with increasing glia signal fraction, though the rate of change is generally greater for the SM than the SV. A disparity is still largely present between the SM and SV under simple axon morphologies at any non-negligible glia signal fraction, suggesting that glia can, indeed, affect the consistency of axon diameter mapping using both the SM and SV. For the simulations with realistic astrocytes, we further show that it is the large astrocytic processes, rather than the soma, which contributes to the overall measured effective MR radius.

## 4 Discussion

Axon diameter mapping currently relies on the assumption that strongly diffusion-weighted signals are specific to axons in the human white matter. The previous observation of a b^-1/2^ scaling suggests that dMRI signal in the high *b* regime comes from water confined inside “sticks” representing impermeable cellular processes with a negligible radius (Veraart et al., 2019). While neuronal projections, or axons, and glial processes share “stick”-like morphological characteristics, the glial contribution was ignored or deemed negligible in the subsequent development of strategies for axon diameter mapping. By testing the consistency of axon diameter mapping under varying experimental factors, we demonstrated that axon diameter mapping is confounded by high *b* signal contributions that are currently not accounted for in the single compartmental model. Monte Carlo simulations further demonstrate that the observation cannot be explained by the presence of spherical cell bodies, but that glial processes are a plausible candidate.

For glial cells to bias axon diameter mapping in the observed manner, there are 5 conditions that must be met: (a) significant volume fractions of glial cells and their processes in the white matter, (b) more isotropically dispersed processes relative to the axons (c) thick processes relative to axons, (d) sufficiently long T2 to result in an observable signal fraction, and (e) sufficiently slow water exchange across its membrane. In addition to our own Monte Carlo simulations, we identify the following corroborating evidence in the literature. First, volume fractions of glial processes have been quantified in (i) the optical nerve, measuring fractions as high as 20% for astrocytes and oligodendrocytes combined (Perge et al., 2009; Walhovd et al., 2014). Coelho et al. (2018) further concluded that the volume of astrocytic processes is substantial (36.4%) relative to the volume of myelinated axons in the human deep subcortical white matter. Second, PLI and microscopy data have shown that the processes of glial cells are more radially dispersed, even in the white matter (Mollink et al., 2017). Third, microscopy data show that the processes of glial cells have large radii relative to axons, particularly in humans (Oberheim et al., 2009; Walhovd et al., 2014). This finding is further supported by evidence from diffusion-weighted MR spectroscopy (Palombo et al., 2017). The remaining conditions (long T2 and slow exchange) are more challenging to validate independently. However, in vitro experiments have shown that the exchange time of glial cells such as astrocytes is higher than their axonal counterpart, but not sufficiently short to be considered permeable within applied diffusion times (Pfeuffer et al., 1998; Yang et al., 2017). However, uncertainty still exists with respect to exchange times of glial cells under biological conditions (Pfeuffer et al., 1998; Yang et al., 2017). As well, the exchange time has been found to have a wide range of values in both the cortex and white matter (Jensen, 2023; Li et al., 2022). There is to our knowledge no data on T2 relaxation times of individual cells. However, Pizzolato et al. (2022) showed in a similar set-up that the “dispersed” and “non-dispersed” compartments at high *b* have distinct T2s, with the dispersed compartment having a T2 of approximately 30 ms (Pizzolato et al., 2022). Such observation is in agreement with clinical studies that reported a reduced T2 relaxation time of glial cells, astrocytes in particular, which was lower than axons due to the glial fibrillary acidic protein polymers and the intermediate filaments (Alshelh et al., 2018; Justicia et al., 2008; Schwarz et al., 1996).

Only a handful of tailored experiments have been performed with the directed purpose of investigating the contribution of glial cells to the dMRI signal. One such study found that the signal fraction of the extra-neurite compartment followed the expected temporal dynamics of reactive microglia activation, induced via a dorsal root axotomy in rats (Taquet et al., 2019). Furthermore, the extra-neurite signal fraction was found to correlate with a histological measurement of microglia density. Convergent on that result, orientation dispersion (OD) measured from a biophysical compartment model was found to correlate with a depletion and subsequent repopulation of brain microglia in rats following CSF1R inhibition (drug causing microglia depletion; Yi et al., 2019). Monte Carlo simulations further demonstrated a positive correlation of OD to extra-neurite space occupancy, and immunofluorescence revealed a positive correlation between OD and microglia density (Yi et al., 2019). A more recent study provides evidence of the potential specificity of the dMRI signal to astrocyte and microglia populations (Garcia-Hernandez et al., 2022). Using rat models of neuroinflammation, degeneration, and demyelination, Garcia-Hernandez et al. (2022) found specific signatures relating to each experimental condition using a bespoke biophysical model including parameters ostensibly related to astrocytes and microglia. They found that their framework was sensitive and specific to inflammation with and without neurodegeneration and demyelination, suggesting that specific glia signatures are present and can be parsed in the dMRI signal. Using dysmyelinated and immunodeficient shiverer mice, Lyczek et al. (2017) found changes in the fractional anisotropy (FA) and radial diffusivity (RD) between the shiverer mice, and mice transplanted with glial restricted progenitors (both human and mouse origin). This suggests that the production of remyelinating oligodendrocytes (and astrocytes) is detectable using dMRI. In the current study, we found divergence of effective MR radius estimates between SM and SV, suggesting that at high *b*-values the signal contributions are not exclusively from neuronal processes. Using Monte Carlo simulations, we confirm that such divergence can be explained by the presence of glial processes (microglia, astrocytes, and oligodendrocytes) at non-negligible signal fractions. While our MR experiments lack specificity, the results here suggest that glia, most likely astrocytes (Walhovd et al., 2014), might contribute to the dMRI signal, particularly at high *b*-values.

Our validation strategy is built upon the presumption that axon diameter mapping must be robust to the variations in the order of the SH representation of the underlying signal. Indeed, the effective MR radius can be derived from either the 0th or 2nd-order RISH features. Minimal adjustments to the forward model are required, but they do not represent additional or alternative modeling assumptions. The adjustment creates a dependency of the effective MRI radius on $D_{c}$. The estimation of $D_{c}$ requires additional data to estimate such parameters through additional biophysical modeling approaches (e.g. SMI). We confirmed that this dependency is not causing a spurious discrepancy between SM- or SV-derived effective MR radii. Indeed, we evaluated for which $D_{c}$ or $D_{0}$ the SM- or SV-derived effective MR radii become indifferentiable. Yet, we could not identify plausible values of $D_{c}$ or $D_{0}$ to achieve that condition. We further note that SV is proportional to *p*_2,_ a metric of intra-cellular coherence, but a Spearman Correlation analysis showed that *p*_2_ does not fully explain the observed discrepancies between SM and SV-based analyses (Supplementary Fig. S2).

By assigning cell-specific labels to biophysical model parameters, for example, axon diameter, or intra-axonal fraction, there is a risk of users biasing their interpretation of such models to such cells, thereby overlooking the potential role of other cell types or processes. This is particularly relevant for glial cells, which are known to be a collection of cells with diverse functions in maintaining brain health. Previously, some research teams have already discussed the potential contribution of glial cells to the *stick* compartment (Jelescu et al., 2020; Nilsson et al., 2013). However, such considerations are not generally adopted as experimental data in support of such a hypothesis has been limited (Alexander et al., 2019). Following our results, further validation studies are needed to support claims regarding cellular-specificity of modeling parameters, or lack thereof.

Some biophysical models have previously included an isotropic compartment of restricted diffusion as a representation of glial cells (Alexander et al., 2010; Palombo et al., 2020; Stanisz et al., 1997). In this study, we identify the isotropically-distributed processes of glial cells to be a more likely contributing factor in the modeling of diffusion signals at high *b* than cell bodies (Walhovd et al., 2014)*.* Further studies are needed to evaluate whether these processes have a unique signature on the signal. If, for example, their intracellular diffusivities, exchange times, or relaxation times (Pizzolato et al., 2022) are sufficiently different, then there is an avenue to model such a glial compartment in the next generation of biophysical models.

We have introduced a new estimator of the effect MR radius of cellular processes. We here propose a log-linear estimator that can be applied with a minimum of two distinct high *b* shells. Compared to the previous estimator, this estimator has a closed-form solution and can be implemented using common tools for arithmetics of images or tabulated values. The log-linear estimator assumes that the Neumann equation of restricted diffusion cylinder is an adequate model, thereby assuming that δ ≫ r^2^/D_0_ (Neuman, 1974). The difference between MR effective radii estimated using the log-linear estimator and the estimator proposed in Veraart et al. (2020), which uses the Van Gelderen method, was on average less than 1.81% for both $r_{SM}$ and $r_{SV}$ . Note that extracting the RISH features from the DW signals still requires a decomposition of the DW signal in the spherical harmonics basis using a bias-correcting approach; for example, Maximum Likelihood estimators. The log-linear estimator builds upon the power law ratio of Pizzolato et al. (2023) and further provides an avenue for the interpretation of Temporal Diffusion Ratio (TDR) (Warner et al., 2023). In TDR, the ratio of two spherically-averaged dMRI signals, each collected with high *b*-value but with a different set of diffusion gradient parameters (e.g., gradient magnitude), has previously shown to have strong correlations with axon diameter. Here, we show that the logarithm of such a ratio is proportional to $r^{4}$.

Our imaging protocol was extensive relative to the minimal requirement for the estimation of the effective MR radius. While only two distinct high *b*-values are necessary for the mapping of the effective MR radius, we also acquired (a) low b-shell data for noise map estimation in support of Rician bias correction and (b) Multishell data with limited gradient strength to enable the estimation of D_c_ using the standard model.

Both imaging protocols and tract-based analysis strategies were designed to maximize the SNR of RISH features, even at such high b-values. When using a voxelwise approach or working with data that is less rich in terms of gradient directions, one must consider the following SNR-dependent confounds in comparing SM- and SV-derived model parameters. First, the estimation of SV is subject to a positive bias that depends on the underlying value of SV and the noise level of estimated SH coefficients. By computing the euclidean norm over five second-order SH coefficients, the resulting SV follows a noncentral Chi distribution with an expectation value that exceeds the underlying value at low SNR (París et al., 2025). Therefore, unlike SM, the accuracy of SV depends not only on the baseline SNR of the imaging data, but also the number of gradient directions for the corresponding b-value and geometry of underlying microstructure. We evaluated how this noise dependence might contribute to differences between $r_{SM}$ and $r_{SV}$ estimates in various SNR regimes (Supplementary Fig. S3). The log-linear estimator might further have a reduced accuracy for both $r_{SM}$ and $r_{SV}$ due an SNR-dependency of the expectation value of a log-transformed variable, a known effect that was previously used to compensate for Rician biases in DTI analyses (Veraart et al., 2013). The bias of log-transformed variables scales as 1 /SNR^2^, and therefore only noticeable at a low SNR of the RISH features. A direct comparison between loglinear and previously used nonlinear estimators showed negligible impact of such confounding factors on our study findings, <1.47%, but they might be relevant in experiments performed in lower SNR regimes. With a baseline SNR in the range of 25-50 per voxel, SNR values of 100 or greater are representative for bundle-averaged segments, mitigating the aforementioned effects of lower SNR.

This study has several limitations that must be considered. First, our study shows that diffusion-weighted signal at high *b* is not specific to a single compartment of sticks, it does not provide evidence of the second compartment being glial cell processes. Dedicated follow-up experiments that measure the impact of active and passive water exchange between glial cells and the extra-cellular on axon diameter mapping will be of importance to confirm the hypotheses that are generated by the presented work. Second, our analysis was limited to the cortico-spinal tract to improve the sensitivity of the analysis. Indeed, the precision of the estimators improves with larger axons that have been observed in the CST. The CST is relatively coherent across its length which also promotes the precision of along-tract profiling, particularly when using the SV which has an SNR inversely proportional to dispersion. However, we confirmed the generalizability of the findings in other tracts (Supplementary Fig. S1). Finally, the observations will likely impact the interpretation of other multi-compartment modeling strategies, including NODDI (Zhang et al., 2012), SMI (Novikov, Veraart, et al., 2018), or SANDI (Palombo et al., 2020). However, such assessments are the subject of future studies.

In agreement with Pizzolato et al. (2023), the current work shows that axon diameter measurements vary with RISH order. This suggests that non-axonal cellular sources may be confounding measurements of axon diameter derived from such high *b*-value signals. We further hypothesize that glial processes might explain the discrepancy, but dedicated experiments are needed to confirm the impact of glial cells on axon diameter mapping with more specificity. Our results underscore the need for caution in the naming and interpretation of axon diameter mapping from diffusion MRI.

## Supplementary Material

Supplementary Material

## Data Availability

The data from this study are available from the corresponding author upon reasonable request. The code for axon diameter mapping will be released on https://github.com/NYU-DiffusionMRI/AxonRadiusMapping.
