# Supplementary material for: Revisiting the interpretation of axon diameter mapping using higher-order signal representations
Source: Imaging Neurosci (Camb). 2026 Jan 9;4:IMAG.a.1080. doi: 10.1162/IMAG.a.1080 (PMC12794307; doi:10.1162/IMAG.a.1080)
Supplement: Supplementary Material [file IMAG.a.1080_supp.pdf]

Revisiting the interpretation of Axon diameter mapping using higher-order signal representations

Bradley G. Karat[1,2], Jamie Wren-Jarvis[3], Erika P. Raven[3,4], Ali R. Khan[1,2], Derek K. Jones[5], Marco Palombo[5,6], Jelle Veraart[3*]

[1]Robarts Research Institute, Western University, London, ON, Canada; [2]Centre for Functional and Metabolic Mapping, Western University, London, ON, Canada; [3]Center for Biomedical Imaging, Department of Radiology, NYU Grossman School of Medicine, New York, NY, United States; [4]Institute for Translational Neuroscience, NYU Grossman School of Medicine, New York, NY, United States; [5]Cardiff University Brain Research Imaging Centre (CUBRIC), School of Psychology, Cardiff University, Cardiff, United Kingdom; [6]School of Computer Science and Informatics, Cardiff University, Cardiff, United Kingdom

*Corresponding author: Jelle Veraart, Jelle.Veraart@nyulangone.org

This PDF file includes:

Figures S1 to S3

While we primarily focus on the CST throughout the work, we here address the generalizability of the findings to other WM tracts, including anterior thalamic radiation (ATR), arcuate fascicle (AF), Uncinate fascicle (UF), Inferior longitudinal fascicle (ILF), cingulum, anterior midbody of the corpus callosum (CC), Middle cerebellar peduncle (MCP), and Superior longitudinal fascicle I/II (SLF). In Supplementary Figure 1, we show the subject-averaged r_{SM} and r_{SV}, and their between-subject variability, along each of the tracts. Trends from both hemispheres are shown when applicable. A total of 1568 segments were compared, thereby covering 16 tracts. Following a paired t-test, we conclude that in 68% all segments r_{SM} and r_{SV} are statistically different (p<0.05). Even when controlling the false discovery rate using Benjamini-Hochberg procedure, 56.9% of the segments are significant. Statistically significant differences were retrieved in all evaluated tracts.

In supplementary figure 2 we evaluate the impact of dispersion on the observed discrepancies between SM and SV-derived axon diameters. We estimate $p_!$ in each of the tract segments using Standard Model Imaging. We then quantify the Spearman correlation (ρ) between $p_!$ and the relative difference between SM and SV. In the left CST we find that ρ=0.14 and in the right CST ρ=0.37. Overall, we conclude that there is only a weak correlation, which does not substantially explain the observed differences in effective MR radius between SM and SV.

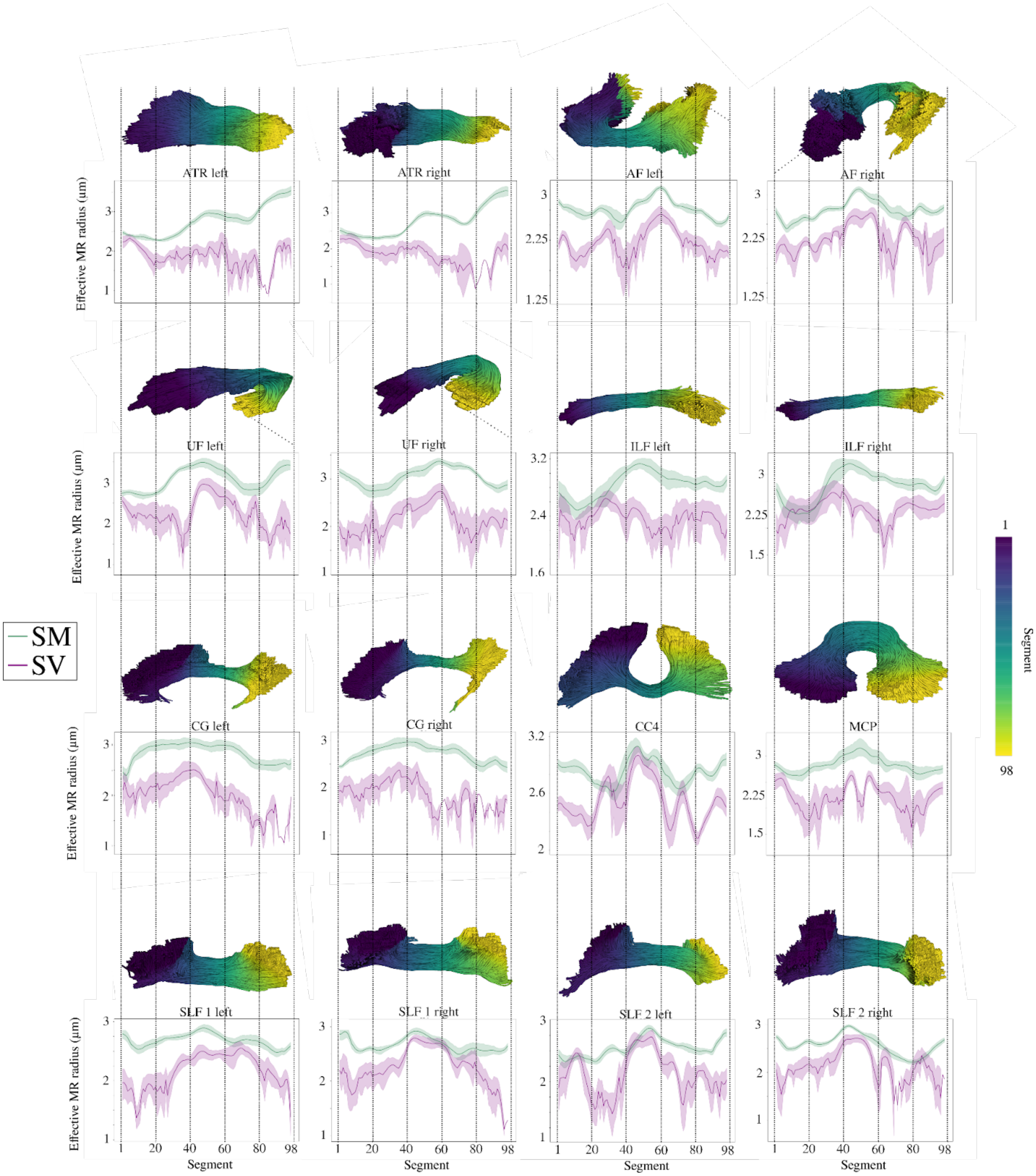

Supplementary Figure 1. Characterization of the effective MR radius across subjects per segment across 16 different WM tracts. Each tract is split into 98 segments, as seen by the colour coding of the reconstructed bundles. The mean of the effective MR radius computed from the spherical Mean (SM) is marked with the solid green line and the spherical variance (SV) is marked with the solid purple line. The 95% confidence interval (CI) is included for both the SM and SV plots.

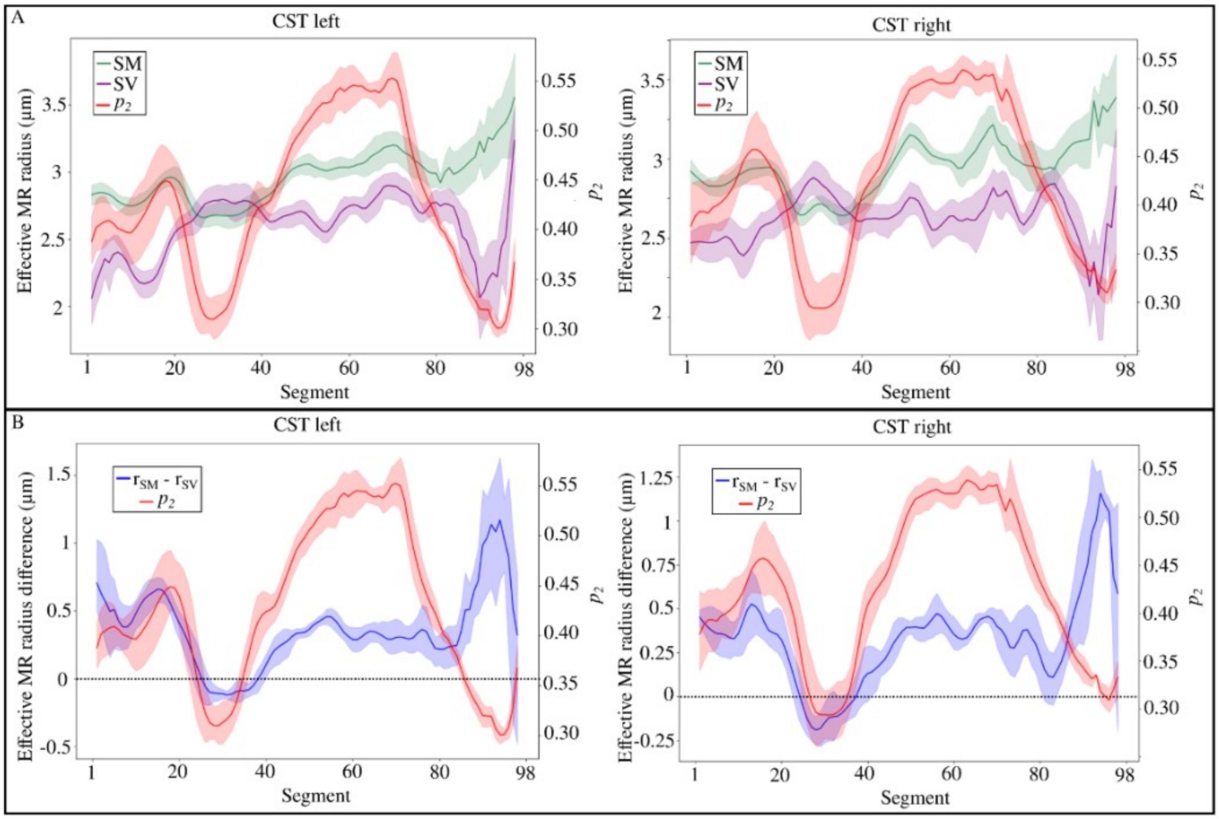

Supplementary Figure 2. (A) Effective MR radius across subjects per segment in the left and right cortico-spinal tract (CST) using the spherical mean (SM) and spherical variance (SV), as well as p_2 from standard model imaging. (B) The difference in effective MR radius between SM and SV and p_2. Spearman's ρ was used to quantify the relationship between the SM-SV difference and p_2 across all segments.

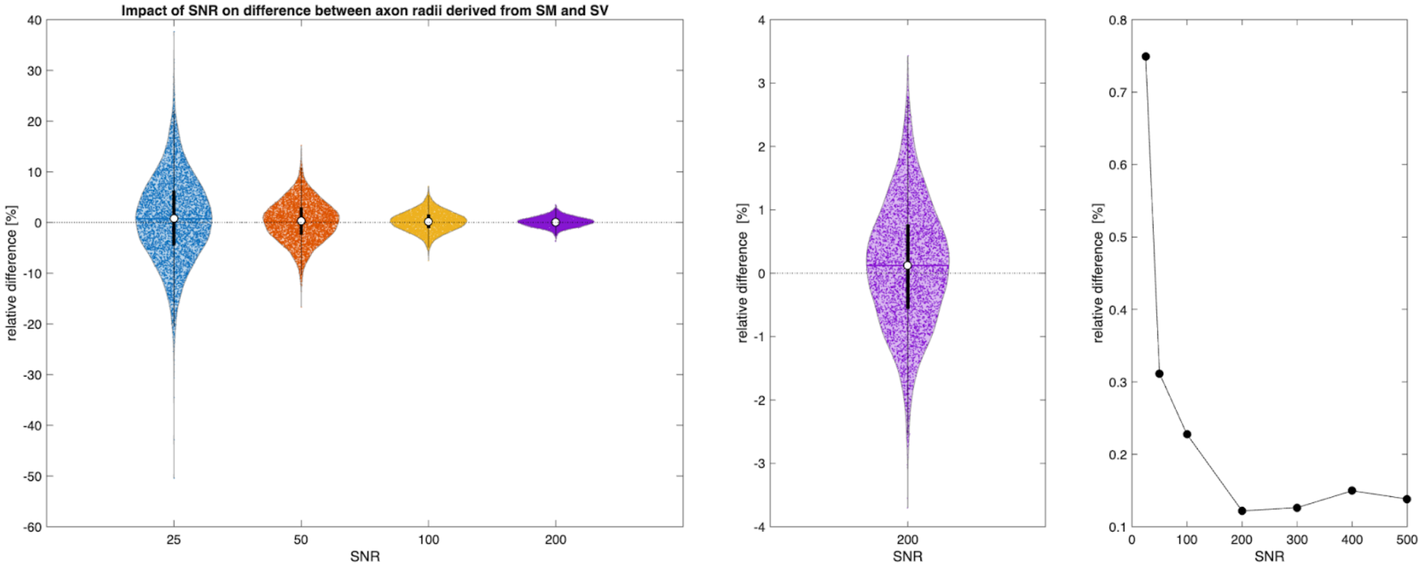

Supplementary Figure 3. The impact of SNR (relative to b0) on the relative difference between axon radii derived from SM and SV for varying SNR using simulated data (5000 noise realizations and matched protocols to in vivo experiments; left). While the SNR range 25-50 is representative for a single voxel, values of 100 and above are representative for bundle-averaged segments. The loglinear estimator was used. We zoom in on the SNR=200 case to demonstrate the shape of distribution and show the magnitude of the effects (middle). The median of the relative differences is shown as a function of SNR - showing errors of 0.2% for representative SNR ranges in our segment-based analysis (right).
